# BML-265 and Tyrphostin AG1478 Disperse the Golgi Apparatus and Abolish Protein Transport in Human Cells

**DOI:** 10.3389/fcell.2019.00232

**Published:** 2019-10-11

**Authors:** Gaelle Boncompain, Nelly Gareil, Sarah Tessier, Aurianne Lescure, Thouis R. Jones, Oliver Kepp, Guido Kroemer, Elaine Del Nery, Franck Perez

**Affiliations:** ^1^Dynamics of Intracellular Organization Laboratory, Institut Curie, PSL Research University, Sorbonne Université, Centre National de la Recherche Scientifique, UMR 144, Paris, France; ^2^BioPhenics High-Content Screening Laboratory, Cell and Tissue Imaging Facility (PICT-IBiSA), Institut Curie, PSL Research University, Translational Research Department, Paris, France; ^3^Equipe Labellisée par la Ligue Contre le Cancer, Université de Paris, Sorbonne Université, INSERM U1138, Centre de Recherche des Cordeliers, Paris, France; ^4^Metabolomics and Cell Biology Platforms, Gustave Roussy, Villejuif, France; ^5^Suzhou Institute for Systems Medicine, Chinese Academy of Medical Sciences, Suzhou, China; ^6^Pôle de Biologie, Hôpital Européen Georges Pompidou, AP-HP, Paris, France; ^7^Department of Women’s and Children’s Health, Karolinska University Hospital, Karolinska Institute, Stockholm, Sweden

**Keywords:** golgi, membrane trafficking, high-content screening, EGFR kinase inhibitor, GBF1

## Abstract

The steady-state localization of Golgi-resident glycosylation enzymes in the Golgi apparatus depends on a balance between anterograde and retrograde transport. Using the Retention Using Selective Hooks (RUSH) assay and high-content screening, we identified small molecules that perturb the localization of Mannosidase II (ManII) used as a model cargo for Golgi resident enzymes. In particular, we found that two compounds known as EGFR tyrosine kinase inhibitors, namely BML-265 and Tyrphostin AG1478 disrupt Golgi integrity and abolish secretory protein transport of diverse cargos, thus inducing brefeldin A-like effects. Interestingly, BML-265 and Tyrphostin AG1478 affect Golgi integrity and transport in human cells but not in rodent cells. The effects of BML-265 are reversible since Golgi integrity and protein transport are quickly restored upon washout of the compounds. BML-265 and Tyrphostin AG1478 do not lead to endosomal tubulation suggesting that, contrary to brefeldin A, they do not target the *trans*-Golgi ARF GEF BIG1 and BIG2. They quickly induce COPI dissociation from Golgi membranes suggesting that, in addition to EGFR kinase, the *cis*-Golgi ARF GEF GBF1 might also be a target of these molecules. Accordingly, overexpression of GBF1 prevents the effects of BML-265 and Tyrphostin AG1478 on Golgi integrity.

## Introduction

The Golgi apparatus lies at the center of the secretory pathway of eukaryotic cells. This organelle receives neo-synthesized secretory proteins from the ER and sorts them to their destination compartments, such as the endosomes or the plasma membrane (Boncompain and Weigel, 2018). In the Golgi complex, cargos are often processed and modified through proteolysis and glycosylation. Defects in the function and/or localization of Golgi associated proteins regulating trafficking and especially of glycosylation enzymes could lead to diverse diseases (Zappa et al., 2018). Golgi-resident glycosylation enzymes are type II transmembrane proteins transported from the ER to the Golgi in a COPII-dependent manner (Ward et al., 2001). The Golgi apparatus is a dynamic polarized organelle the composition of which is controlled by a balance of anterograde and retrograde fluxes of proteins. In particular, the steady-state intra-Golgi localization of Golgi-resident glycosylation enzymes is ensured by constant recycling from cisterna to cisterna up to the ER (Storrie et al., 1998). Retrograde transport of glycosylation enzymes occurs via COPI vesicles either by direct binding to coatomer (Liu et al., 2018) or through interaction with GOLPH3 (Tu et al., 2008; Ali et al., 2012). COPI coat recruitment is modulated by ADP-ribosylation factors (ARF), which cycle from an inactive state (GDP-bound) to an active state (GTP-bound) (Serafini et al., 1991; Donaldson et al., 1992a). ARFs are activated by guanine nucleotide exchange factors (GEF). At the *cis*-Golgi, the Golgi brefeldin A-resistant factor 1 (GBF1) plays a pivotal role in regulating organelle structure and vesicle trafficking by catalyzing the activation of ARF1 leading to COPI assembly and recruitment to Golgi membranes (Presley et al., 2002).

Because anterograde transport of cargos, especially of Golgi-resident glycosylation enzymes, is constantly counterbalanced by retrograde transport (Cole et al., 1996; Storrie et al., 1998; Sengupta et al., 2015), it is difficult to distinguish between defects in the anterograde or the retrograde transport when a Golgi enzyme localization is perturbed. The development of the Retention Using Selective Hooks (RUSH) assay enables to overcome this difficulty through synchronization of fluxes, enabling quantitative analysis of anterograde transport of cargos (Boncompain et al., 2012). The RUSH assay uses physiological conditions for mammalian cells and is induced by simple addition of the non-toxic vitamin biotin. We have previously shown that the RUSH assay is amenable to high-content phenotypic screening (Boncompain et al., 2012, 2019; Liu et al., 2017; Zhao et al., 2018). To identify small molecules that regulate the trafficking of Golgi-resident glycosylation enzymes, we combined the RUSH assay to high content screening of chemical libraries using Mannosidase II (ManII) as a model Golgi enzyme. We identified several molecules either inhibiting or accelerating ManII transport from ER to Golgi. Supervised classification enabled the discovery of two small molecules displaying BFA-like activity on which we focused the present study. We further showed that these molecules, namely BML-265 and Tyrphostin AG1478, described as EGFR kinase inhibitors have additional intracellular biological effects. Both compounds induced reversible Golgi disruption and inhibition of the secretory transport in human cells, but not in rodent cells. The analysis of their effects on endosomal tubulation and dissociation of COPI from Golgi membranes suggested the *cis*-Golgi ARF GEF GBF1 to be the target of BML-265. This was further supported by overexpression of human GBF1, which prevents the effects of BML-265 and Tyrphostin AG1478.

## Materials and Methods

### Screening Procedure

#### Cell Seeding

HeLa cells stably expressing Streptavidin-KDEL_ManII-SBP-EGFP (Boncompain et al., 2012) were counted using T4 Cellometer (Nexcelom) and seeded in 384-well plates (ViewPlate-384 Black Perkin Elmer, catalog number 6007460) at 2,000 cells/well using a Multidrop Combi (Thermo Fisher Scientific) in 40 μl of cell media. The screen was performed at same early cell passages in two replicate experiments.

#### Compound Libraries

A FDA-approved drug library, comprised of 640 compounds diluted in Dimethyl Sulfoxide (DMSO) was purchased from Enzo Life Sciences (BML-2842) together with 80 known kinase inhibitors of well-defined activity (Screen-Well Kinase Inhibitor Library, catalog number BML-2832), 33 phosphatase inhibitors (Phosphatase Inhibitor library, catalog number BML-2834) and 53 protease inhibitors (Screen-Well^TM^ Protease Inhibitor Library, catalog number BML-2833). 24 h after seeding, 10 μL of compounds was transferred to 384-well plates using the MultiChannel Arm^TM^ 384 (MCA 384) (TECAN) to the cells, to a final concentration of 10 μM and 0.5% of DMSO. Nocodazole (2.5 μM final dilution) and brefeldin A (10 μM final dilution) were added as biologically relevant phenotypic controls in a single control plate. All chemical compounds were diluted in dimethyl sulfoxide (DMSO) as 10 mM stock solution and robotically reformatted in-house into 384-well source plates.

#### Compound Treatment and Biotin Pulse

After 90 min of incubation with the compounds at 37°C and 5% CO_2_, cells were treated with 40 μM of biotin for 30 min at 37°C. The screening was performed in three biological replicates. After biotin treatment, cells were processed for immunofluorescence performed as follows: cells were fixed with a fresh solution of 3% paraformaldehyde for 15 min using the MultiChannel Arm^TM^ 384 (MCA 384) (TECAN). Cells were quenched with 50 mM NH_4_Cl in phosphate buffered saline (PBS) solution for 5 min, and then incubated for 1 h at room temperature (RT) with a primary mouse anti-GM130 antibody (1:500, BD Biosciences, catalog number 610822) diluted in 1% BSA-0.2% saponin. Cells were further washed with PBS and co-incubated for 1h RT with a secondary anti-mouse Alexa 647 antibody (1:400, Thermo Fisher Scientific, catalog number A-31571) and Hoechst 33242 (DNA) (1:500, Sigma, catalog number 14533).

For dose-response experiments, cells were treated 24 h after seeding with compounds (20 μL of media/DMSO solution) and vehicle DMSO 0.5%, titrated in a 8- point, three-fold dilution starting at a concentration of 10 μM. The assay plates were then incubated 24 h at 37°C at 5% CO_2_ and processed as described above.

### Image Acquisition and Analysis

Image acquisition was performed using the INCell 2000 automated widefield system (GE Healthcare, United States) at a 20X magnification (Nikon 20X/0.45, Plan Apo, CFI/60), using the same exposure time for all plates in the experiment and across replicate experiments. Plates were loaded onto the microscope system with a Kinedx robotic arm (PAA, United Kingdom). Images of four different positions in each well were acquired, each containing channels for Hoechst 33242, ManII-SBP-EGFP and GM130. The total number of cells measured in a well was typically around 300. Computational image processing operations were performed using the dual area object analysis in the INCell Analyzer Workstation 3.7 software (GE Healthcare). Nuclei were defined based on DNA staining and cell region was segmented using top-hat and collar segmentation, respectively. ManII-SBP-EGFP in the Golgi area was identified as inclusions in the collar cell area using multiscale top-hat segmentation, and quantified by the average intensity of pixels within the defined inclusion region. Individual cells were then gated to single phenotypes using CellProfiler Analyst software (Jones et al., 2008). Briefly, cell samples from all replicate experiments were sorted into four-defined classes (1. Golgi-disrupted, 2. ER + Golgi, 3. ER-retained and 4. Golgi) using DMSO, brefeldin A and nocodazole-treated wells as a training set for the classifier active learning module. Obtained data was normalized using the robust Z-score method under the assumption that most compounds are inactive and can serve as controls (Malo et al., 2006; Birmingham et al., 2009). Plate positional effects were corrected using median polishing (Mosteller and Tukey, 1977; Birmingham et al., 2009) applied to each phenotypic class. Hits for each compound were identified as follows: sample median and median absolute deviation (MAD) were calculated for each replicate from the population of screening data points (named as sample) and used to compute Robust Z-scores [RZ-scores (Iglewicz and Hoaglin, 1993)] according to the formula: RZscore = (Perturbator value-median (reference sample))/(1.4826 × MAD). A compound was identified as a ‘hit’, if the RZ-score was <−2 or >2.

### Cells, Plasmids, Transfection

Hela wildtype and stably expressing Str-KDEL_ManII-SBP-EGFP (Boncompain et al., 2012) cells, mouse embryonic fibroblasts (MEF) and normal rat kidney (NRK) cells were cultured in Dubelcco’s modified Eagle medium (DMEM) (Thermo Fisher Scientific) supplemented with 10% Fetal Calf Serum (FCS, GE Healthcare), 1 mM sodium pyruvate and 100 μg/ml penicillin and streptomycin (Thermo Fisher Scientific).

RUSH plasmids were previously described TNF-SBP-EGFP, SBP-EGP-GPI (Boncompain et al., 2012), SBP-EGFP-EGFR (Scharaw et al., 2016).

The plasmid coding for Venus-GBF1 was a kind gift from C. Jackson (Institut Jacques Monod, Paris, France).

HeLa cells were transfected using calcium phosphate as described previously (Jordan et al., 1996).

### Chemicals

BML-265 was purchased from Enzo Life Sciences. Tyrphostin AG1478 and Erlotinib were ordered from Cayman chemical. DMSO, brefeldin A, Golgicide A and D-biotin were purchased Sigma-Aldrich.

### Antibodies and Immunofluorescence

Monoclonal mouse anti-GM130 was purchased from BD Biosciences (catalog number 610823, used at 1:1000) and rabbit polyclonal anti-GM130 from Abcam (catalog number ab52649, dilution 1:2000). Anti-GFP was purchased from Merck (catalog number 11814460001, use at 1:1000). Anti giantin (TA10) was obtained from the recombinant antibody platform of the Institut Curie (dilution 1:100). Anti betaCOP (mAD) and anti-transferrin receptor (OKT9) were a gift from T. Kreis, University of Geneva, dilution 1:200 and 1:1000 respectively. The anti-betaCOP antibody requires cell fixation using methanol. The antibody directed to GalT (B4GalT1) used in Supplementary Figure S2 was purchased from Abnova (catalog number PAB20512, dilution 1:1000) and the one used in Supplementary Figure S3 was obtained from CellMAb (catalog number CB02, dilution 1:100). The anti-TGN46 was purchased from BioRad (catalog number AHP500G, dilution 1:2000). The antibody directed to Sec24C was a kind gift from David Stephens (University of Bristol, United Kingdom) (dilution 1:500). The anti-EEA1 antibody was purchased from BD biosciences (catalog number 610456, dilution 1:2000). The anti-LAMP1 was purchased from Merck (catalog number L1418, dilution 1:3000).

Alexa488, Cy3 and Cy5 conjugated secondary antibodies were purchased from Jackson Immunoresearch and used at 1:400.

Immunostaining of intracellular targets was performed after fixation with paraformaldehyde 3% for 15 min at room temperature. After 3 washes with PBS, cells were permeabilized with PBS supplemented with BSA 2 g/l and saponin 0.5 g/l for 5 min at room temperature. Antibodies were diluted in PBS supplemented with BSA 2 g/l and saponin 0.5 g/l and incubated with cells for 45 min. Coverslips were mounted in Mowiol containing DAPI.

Immunostaining to monitor the presence of EGFP-tagged cargos at the cell surface was performed on living cells seeded on glass coverslips. Cells were washed with ice cold PBS and incubated on ice with the anti-GFP antibody diluted in PBS for 45 min. Cells were then washed 3 times with ice cold PBS and fixed with paraformaldehyde 2% for 15 min at room temperature. After 3 washes with PBS, the cells were incubated with the secondary antibodies for 40 min at room temp.

After 3 washes with PBS, coverslips were in Mowiol. Observation and acquisition of pictures of fixed samples were performed using an epifluorescence microscope (Leica) equipped with a Coolsnap camera (Roper Scientific) using the software Metamorph (Molecular Devices).

### EGFR Phosphorylation and Immunoblot

Cells were serum starved overnight. Cells were then pre-treated with the indicated molecules at 10 μM for 1h. They were then stimulated with human EGF at 50 ng/ml final for 10 min.

Cells were lysed in Laemmli 2,5 × buffer containing beta-mercaptoethanol. Samples were then loaded on acrylamide gels (Criterion TGX, BioRad) and transferred on nitrocellulose membrane (GE Healthcare) using Power blotter Semi dry from Thermo Scientific. Blocking and incubation of antibodies were performed in PBS supplemented with 0.1% Tween and 5% milk. Detection was performed using SuperSignal West Pico Substrate from Thermo and Chemidoc machine (BioRad).

Human EGF was purchased from Sigma-Aldrich (catalog number E9644).

Antibodies to detect phosphorylated EGFR and total EGFR were purchased from Cell Signaling Technology (PhosphoPlus EGFR (Tyr1068) Antibody Duet, catalog number 11862S, dilution 1:1000). Anti-actin (clone AC40) used as loading control was purchased from Sigma Aldrich (catalog number A3853, dilution 1:1000). Secondary anti-mouse and anti-rabbit poly-HRP antibodies were purchased from Thermo Scientific (dilution 1:10000).

### Real Time Imaging

HeLa cells stably expressing Streptavidin-KDEL_ManII-SBP-EGFP were grown on 25 mm glass coverslips. Cells were maintained in presence of biotin at 40 μM to allow stable localization of ManII-SBP-EGFP in the Golgi apparatus. Coverslips were transferred to an L-shape tubing equipped Chamlide chamber (Live Cell Instrument). Pre-warmed Leibovitz medium (Life Technologies) supplemented with 40 μM of biotin was used. BML-265 diluted at 10 μM in Leibovitz supplemented with biotin was added at time 0. After the indicated time, BML-265 was washed thanks to several washes with pre-warmed Leibovitz. For the recovery period, medium was replaced by pre-warmed Leibovitz containing biotin. Imaging was performed at 37°C in a thermostat controlled chamber using an Eclipse 80i microscope (Nikon) equipped with a spinning disk confocal head (Perkin) and an Ultra897 iXon camera (Andor). Image acquisition was performed using MetaMorph software (Molecular Devices). Maximum intensity projections of several Z-slices are shown.

## Results

### High-Content Phenotypic Screen Led to Identification of Molecules Regulating the Trafficking of ManII

**FIGURE 1:**
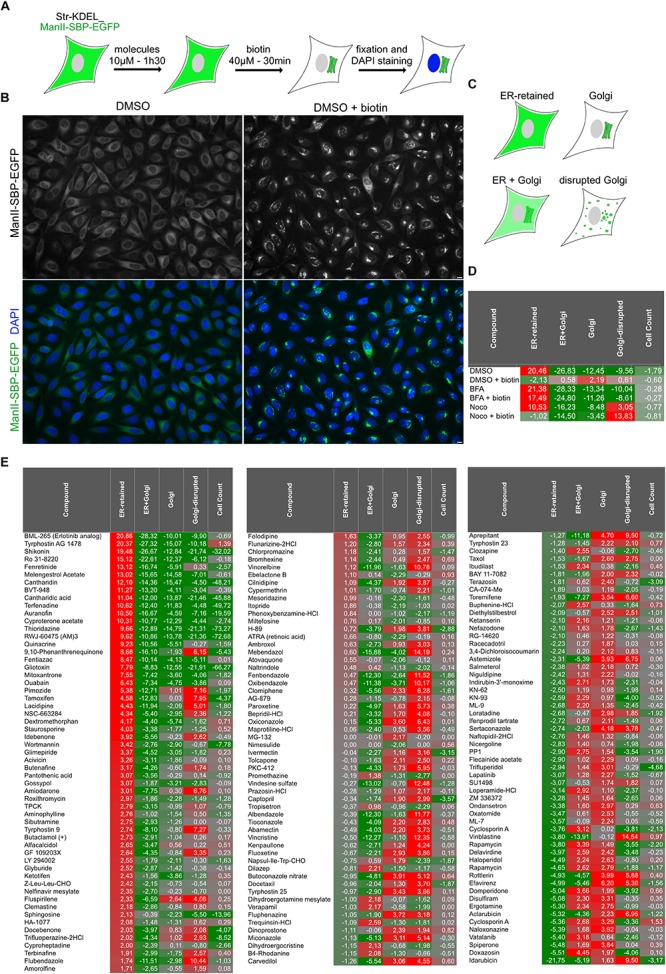
High-content screening for molecules regulating ER to Golgi transport of ManII. **(A)** Scheme of the screening processes. Cells stably expressing Str-KDEL_ManII-SBP-EGFP were incubated with each small molecule for 90 min. Synchronized transport of ManII was induced by addition of biotin. After 30 min, cells were then fixed and nuclei were stained with DAPI. **(B)** Pictures from screening plates depicting the controls DMSO (no traffic) and DMSO + biotin (transport to the Golgi). Scale bar: 10 μm. **(C)** Scheme of the four classes used for the classification of quantitative analysis of the pictures. **(D,E)** Scores obtained for the parameters ‘ER-retained,’ ‘ER + Golgi,’ ‘Golgi,’ ‘Golgi-disrupted,’ and cell count for each control **(D)** or small molecule compound **(E)**.

Using the previously described HeLa cell line stably expressing Streptavidin-KDEL_ManII-SBP-EGFP (Boncompain et al., 2012), we designed a phenotypic screen to identify small molecules modulating the trafficking of the Golgi enzyme Mannosidase II (ManII) (Figure 1A). The cells co-express a non-fluorescent ER hook (Str-KDEL) with ManII fused to a Streptavidin Binding Peptide (SBP) and an EGFP (ManII-SBP-EGFP) enabling RUSH control of ManII trafficking. ManII-SBP-EGFP is retained in the ER in the absence of biotin and is transported to the Golgi apparatus after incubation with biotin (Figure 1B). This set-up was used to screen small molecules from a library of FDA-approved molecules and inhibitors of kinases, phosphatases and proteases (see Materials and Methods section for details). Cells seeded in 384-well plates were incubated with small molecules, diluted at a final concentration of 10 μM in DMSO, for 90 min. Biotin was then added for 30 min to induce the transport of ManII-SBP-EGFP from ER to Golgi. Cells were then fixed and nuclei were stained using DAPI. The Golgi disrupting agent, brefeldin A (BFA) and the microtubule-depolymerizing drug, nocodazole (Noco) were used as positive phenotypic controls for ManII ER-retention and Golgi disruption, respectively. In addition, wells not incubated with biotin were included in order to prevent ManII-SBP-EGFP exit from the ER. 0.5% of DMSO was used as solvent control. After image acquisition and segmentation, several features were measured (see Materials and Methods section for details). Samples were classified into four phenotypical classes (1. ER-retained, 2. ER + Golgi, 3. Golgi disrupted and 4. Golgi) using above-mentioned controls as training set using Cell Profiler Analyst (Figure 1C). A cell count was also used to evaluate toxic effects of the compounds.

Robust z-score for each phenotypic class was calculated (see Materials and Methods) allowing identification of outliers. For instance, the ER-retained robust Z-score of the condition “DMSO” is very different (20.46) from the one of the condition “DMSO + biotin” (−2.19) (Figure 1D). “DMSO + biotin” corresponds to the control condition, for which ER to Golgi transport of ManII occurred normally while “DMSO” (i.e., absence of biotin) is exemplifying the robust Z-score obtained by a hit preventing normal transport and inducing ER-retention. Please note that the score for Golgi-disruption of cells incubated with nocodazole and biotin (‘Noco + biotin’) is higher than the one of cells treated with nocodazole only (‘Noco’) because our analysis uses ManII-SBP-EGFP signal to detect Golgi elements and not an independent Golgi marker. In consequence, the accumulation of ManII-SBP-EGFP in Golgi mini-stacks due to biotin addition leads to a better detection of Golgi-disruption (Figure 1D).

As we were primarily looking for molecules inhibiting ER to Golgi transport of ManII, the results were sorted using ER-retained score (Figures 1D,E). The two top hit molecules identified as inhibitors of ManII ER to Golgi transport were BML-265 and Tyrphostin AG1478. Their score for the 4 phenotypic classes was similar to the controls DMSO [No biotin], BFA and BFA + biotin confirming that they inhibit ManII transport to the Golgi (Figures 1D,E).

We then performed a dose-response analysis, in triplicates, using serial dilutions (10 doses from 30 μM to 1.52 nM) of 50 selected molecules either inhibiting or accelerating ER to Golgi transport. The same quantitative analysis that was done in the primary screen was carried out here. Four families of compounds were identified based on the dose-response profile of ‘ER-retained’ and ‘Golgi disrupted’ scores (Supplementary Figure S1). Some molecules inhibited ER to Golgi transport at increasing doses without affecting the integrity of the Golgi apparatus. These molecules are suspected to affect cell homeostasis or to be energy poisons as this family includes ouabain (Na^+^, K^+^ ATPase inhibitor). Based on the dose-response profiles, we identified two groups of molecules affecting microtubules. A first group contained depolymerizing agents such as albendazole, while a second one was composed of destabilizing agents such as vinca alkaloides (e.g., vinblastine). The dose-response profiles of ‘ER retained’ and ‘Golgi disrupted’ scores showed that BML-265 and Tyrphostin AG1478 displayed similar effects (BFA-like). They inhibited the trafficking of ManII to the Golgi starting from 123 – 370 nM and induced Golgi disruption on the same concentration range. Note that, surprisingly, no Golgi disruption was detected at high doses of BML-265 and Tyrphostin AG1478. This is due to the way we quantified Golgi organization. Indeed, at high doses, the Golgi apparatus was completely disassembled and no visible structures remained. In consequence, our analysis for Golgi disruption did not detect any Golgi structures and scored it as “no Golgi disruption” (Supplementary Figure S1).

### The Effects of BML-265 and Tyrphostin AG1478 on Golgi Integrity and Trafficking Indicate BFA-Like Activity

**FIGURE 2:**
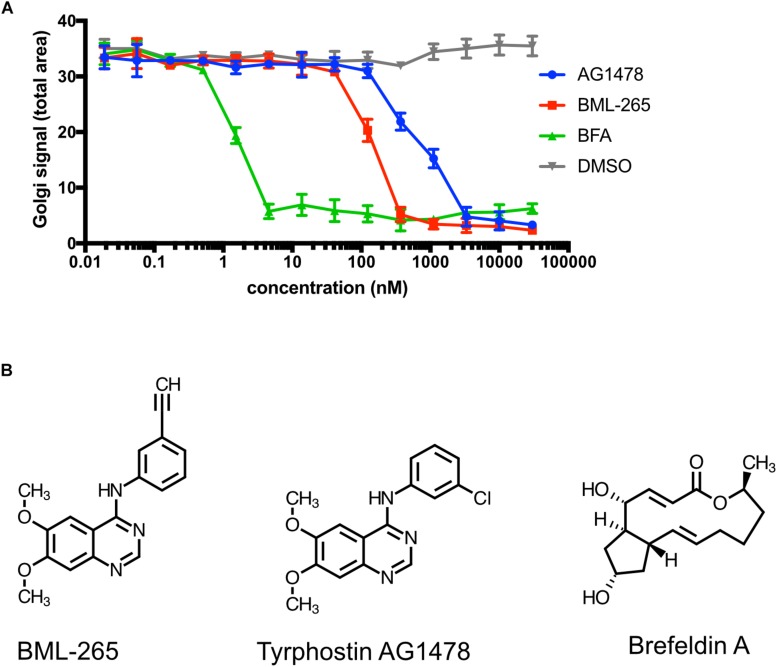
BML-265 and Tyrphostin AG1478 display BFA-like effects. **(A)** HeLa cells stably expressing Str-KDEL_ManII-SBP-EGFP were incubated with serial dilutions of DMSO, BML-265, Tyrphostin AG1478 (AG1478) and brefeldin A (BFA) for 90 min. Cell were then treated with biotin, fixed and stained using an anti-GM130 antibody. ManII-SBP-EGFP signal at the Golgi was then quantified (a.u., arbitrary units) by fluorescence microscopy. 2 independent replicates were performed. Error bars show standard deviation. **(B)** Schemes of the molecules BML-265, Tyrphostin AG1478, and brefeldin A (BFA).

The above-mentioned effects of BML-265 and Tyrphostin AG1478 were reminiscent of the ones obtained with BFA used as a control in our experiments. In addition, whereas BML-265 and Tyrphostin AG1478 are both annotated as EGFR kinase inhibitors, Tyrphostin AG1478 was described to target the *cis*-Golgi ADP ribosylation factor guanine nucleotide exchange factor (ARF GEF) named GBF1 (Pan et al., 2008). We thus decided to compare the efficacy of BML-265, Tyrphostin AG1478 and BFA on the trafficking of ManII to the Golgi apparatus. HeLa stably expressing Str-KDEL_ManII-SBP-EGFP were incubated with serial dilutions of the molecules (from 0.019 nM to 30 μM) for 90 min and biotin was then added to induce the transport of ManII-SBP-EGFP. Our results show that BFA is more potent than BML-265 which is itself more potent than Tyrphostin AG1478 (Figure 2A). Whereas IC50 of BFA is about 2 nM, IC50 of BML-265 is about 200 nM and of Tyrphostin AG1478 about 1 μM. Strikingly, the chemical structures of BML-265 and Tyrphostin AG1478 are closely related while they are different from the one of BFA (Figure 2B).

**FIGURE 3:**
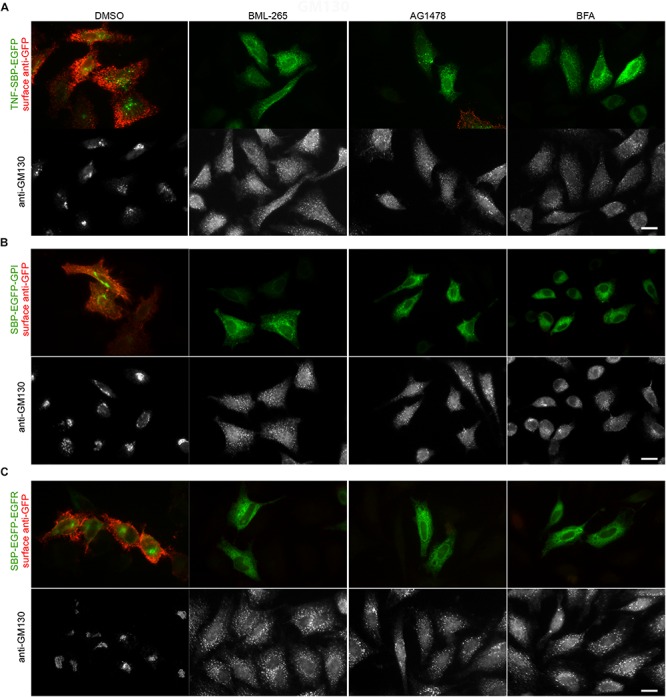
BML-265 and Tyrphostin AG1478 inhibit the transport of secretory proteins. HeLa cells transiently expressing Str-KDEL_TNF-SBP-EGFP **(A)**, SBP-EGFP-GPI **(B)** or SBP-EGFP-EGFR **(C)** were pre-treated with the indicated molecules at 10 μM for 1 h. Trafficking of the reporters was then induced by incubation with biotin for 1 h. The presence of the GFP-tagged reporters (green, upper panel) at the plasma membrane was then detected using an anti-GFP antibody on non-permeabilized cells (red, upper panel). The Golgi apparatus was visualized using immunostaining against GM130 (bottom panel). Scale bar: 10 μm.

We then assessed the effects of BML-265 and Tyrphostin AG1478 on the trafficking of secretory cargos addressed to the cell surface. For this purpose, we used TNF (type II transmembrane protein), GPI (GPI anchor) and EGFR (type I transmembrane protein) as RUSH cargos (Boncompain et al., 2012; Fourriere et al., 2016; Scharaw et al., 2016). In these fusion constructs, the EGFP is exposed to the extracellular space when the cargo reaches the plasma membrane. The effects of the molecules on the anterograde transport of TNF, EGFP-GPI and EGFR were thus assessed by immunofluorescence on non-permeabilized cells using an anti-GFP antibody. The Golgi apparatus was stained using an anti-GM130 antibody. As observed with BFA, pre-treatment of the cells with BML-265 and Tyrphostin AG1478 for 60 min prior to addition of biotin prevented transport of the cargos to the cell surface (Figure 3). The EGFP-tagged cargos are detected inside the cells, probably in the ER. Consistently with the results obtained during the screening and IC50 experiments, the Golgi apparatus is disrupted upon incubation with BML-265 and Tyrphostin AG1478.

Altogether, our results indicate that BML-265 and Tyrphostin AG1478 display BFA-like effects on trafficking and Golgi integrity.

### BML-265 Disperses the Golgi Apparatus in Human Cells but Not in Rodent Cells

**FIGURE 4:**
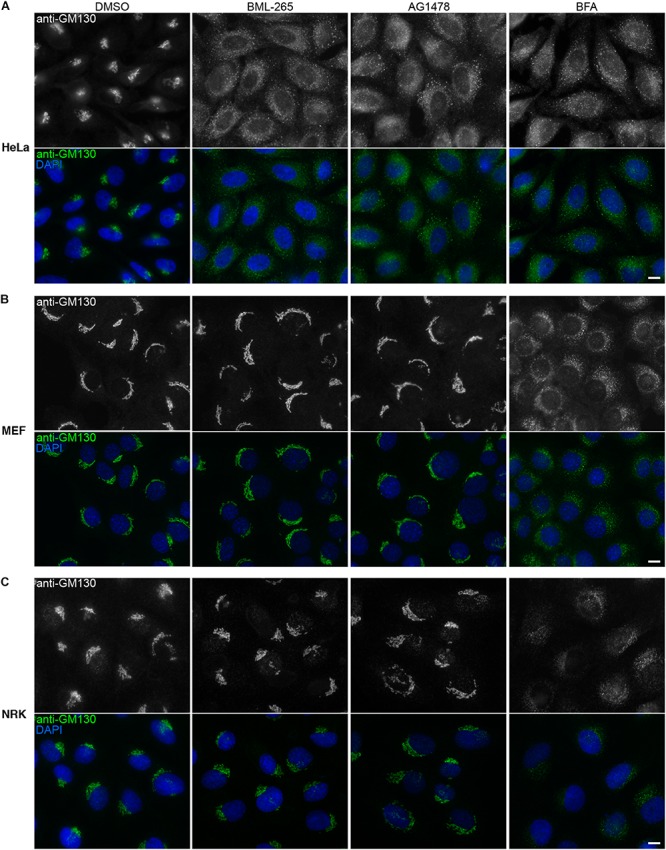
BML-265 affects Golgi integrity in human cells but not in rodent cells. HeLa cells **(A)**, mouse embryonic fibroblasts (MEF) **(B)** or normal rat kidney (NRK) cells **(C)** were incubated with the indicated molecules at 10 μM final for 1h30. Cells were then fixed and the Golgi apparatus was stained using an anti-GM130 antibody (green on merge). Nuclei were stained using DAPI (blue on merge). Scale bar: 10 μm.

Tyrphostin AG1478 was previously reported to affect Golgi integrity in human but not in rodent cells. We thus analyzed the effects of BML-265 on the integrity of the Golgi complex by immunolabeling using an anti-GM130 antibody using a human cell line and two rodent cell lines. As observed with BFA, incubation of the human epithelial cell line HeLa with BML-265 and Tyrphostin AG1478 led to redistribution of GM130, indicating Golgi disruption (Figure 4A). Similar results were obtained when staining for other Golgi markers, namely TGN46, GalT and giantin. As expected, the intracellular distribution of ER exit sites, stained with an anti-Sec24 antibody, was also affected. In contrast, early endosomes and late endosomes/lysosomes organization was not perturbed after incubation with the molecules compared to DMSO control (Supplementary Figure S2). However, these molecules did not affect Golgi localization and morphology of mouse fibroblasts (MEF) and rat epithelial cells (NRK). In contrast, BFA disrupted the Golgi complex of these two rodent cell models (Figures 4B,C).

BML-265 and Tyrphostin AG1478 are both annotated in the library as analogs of Erlotinib (Tarceva trade mark; OSI Pharmaceuticals, Genentech and Roche), one of the several EGFR tyrosine kinase inhibitors, which has been largely studied in clinical trials, with proven efficacy in humans. BML-265, Tyrphostin AG1478 and Erlotinib have different structures but share a quinazoline group (Figure 2B and Supplementary Figure S3). We verified that BML-265 was indeed able to prevent EGFR phosphorylation upon stimulation with EGF. Importantly, Erlotinib did not induce Golgi disruption even at high doses (Supplementary Figure S3). These results suggest that effects of BML-265 and Tyrphostin AG1478 on Golgi integrity and function are independent of their capability to inhibit EGFR phosphorylation.

### BML-265 Has Reversible Effects on Golgi Integrity

**FIGURE 5:**
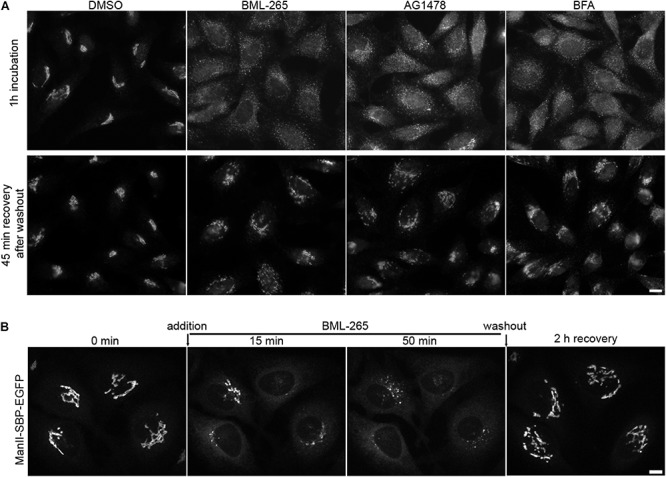
The effects of BML-265 on Golgi integrity are reversible. **(A)** HeLa cells were treated with the indicated molecules at 10 μM for 1h and molecules were removed by extensive washings. Cells were then incubated in normal medium for 45 min, fixed and the Golgi apparatus was stained using an anti-GM130 antibody. Scale bar: 10 μm. **(B)** HeLa cells stably expressing Str-KDEL_ManII-SBP-EGFP were cultivated in presence of biotin 40 μM to allow stable Golgi localization of ManII-SBP-EGFP and imaged by spinning disk confocal microscopy. Under the microscope, BML-265 was added at 10 μM final at time 0 min. After 50 min, BML-265 was washed out and pictures were acquired every 30 s. Maximum projection of 11 z-slices is shown. Scale bar: 10 μm.

Treatment of HeLa cells for 1h with BML-265 or Tyrphostin AG1478 leads to a complete redistribution of Golgi complex proteins throughout the cytoplasm (Figure 5A). However, the effects of both compounds are partially reversible. 1 h after addition of the molecules, the medium was removed and cells were washed before being incubated in normal medium. Cells were then fixed and the Golgi complex immunolabeled using an anti-GM130 antibody. Forty-five min after washout, cells recovered a normal Golgi organization and localization (Figure 5A). We next assessed by real-time imaging the re-formation of the Golgi complex after washout of BML-265. BML-265 was added to HeLa stably expressing ManII-SBP-EGFP. Quickly after addition of BML-265, transient ManII-SBP-EGFP positive tubules were observed and ManII-SBP-EGFP signal intensity at the Golgi decreases while increasing in the whole cell corresponding to ER relocation of ManII-SBP-EGFP (Figure 5B and Supplementary Movie S1). Washout of BML-265 was then performed and cells imaged in real-time. The Golgi complex visualized by ManII-SBP-EGFP redistributed to the perinuclear area. Signal intensity of ManII-SBP-EGFP at the Golgi increased while ER signal decreased showing that ER to Golgi transport was restored.

### BML-265 Might Target GBF1

**FIGURE 6:**
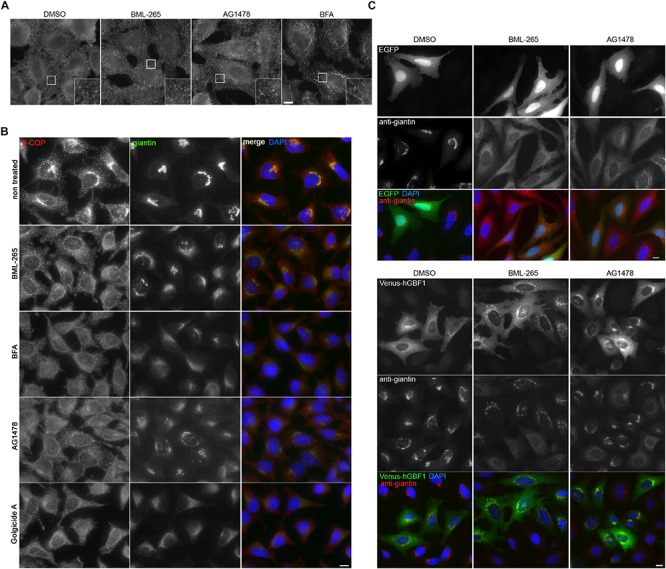
BML-265 exerts its effects on Golgi integrity and trafficking through targeting GBF1. **(A)** HeLa cells were incubated with the indicated molecules at 10 μM for 1 h. After fixation, endosomes were stained using an anti-transferrin receptor (TfR) antibody. Scale bar: 10 μm. The inset displayed an enlarged view of the boxed area. **(B)** HeLa cells were incubated with the indicated molecules at 10 μM for 5 min and were then fixed using methanol. COPI coat was stained using an anti-betaCOP antibody, the Golgi apparatus using anti-giantin antibody and nuclei using DAPI. Scale bar: 10 μm. **(C)** HeLa cells transiently expressing human GBF1 tagged with Venus (Venus-hGBF1) or cytoplasmic EGFP as a transfection control were incubated with the indicated molecules at 10 μM for 1 h. After fixation, the Golgi apparatus was immunolabeled using an anti-giantin antibody. Scale bar: 10 μm.

BFA inhibits Arf1 activation (Donaldson et al., 1992b; Helms and Rothman, 1992) by targeting several ARF GEF: GBF1 at the *cis*-Golgi and BIG1 and BIG2 at the TGN (Claude et al., 1999; Yamaji et al., 2000). Due to its effects on the TGN ARF GEF, BFA induces tubule formation at the TGN and on endosomes (Lippincott-Schwartz et al., 1991; Wood et al., 1991) as confirmed by immunostaining using an anti-Transferrin receptor (TfR) antibody. In contrast, BML-265 and Tyrphostin AG1478 did not induce tubulation of TfR-positive compartments, suggesting that they do not affect the TGN ARF GEF (Figure 6A). GBF1 is an ARF GEF present at the *cis*-Golgi involved in the recruitment of the COPI coat on Golgi membranes. We next assessed the distribution of COPI after treatment with BML-265. In HeLa cells treated with the molecules for 5 min, the distribution of the COPI coat was monitored using immunolabeling with an anti-betaCOP antibody and the Golgi complex was detected using an anti-Giantin antibody. Whereas in non-treated cells, betaCOP is found in the Golgi area, probably associated to the Golgi complex and to vesicles, betaCOP is absent from the Golgi complex in BML-265 treated cells even in cells still displaying a perinuclear Golgi complex after this short treatment time (Figure 6B). As expected, BFA also induced rapid dissociation of COPI coat from Golgi membranes. We also detected dissociation of betaCOP from Golgi membranes for two molecules known as inhibitors of GBF1, Tyrphostin AG1478 and Golgicide A (GCA) (Pan et al., 2008; Saenz et al., 2009) (Figure 6B). Overexpression of human wild-type GBF1 prevented Golgi dispersal induced by incubation with BML-265 or Tyrphostin AG1478 (Figure 6C).

Altogether these results suggest that BML-265 targets GBF1 causing Golgi dispersal and inhibiting secretory protein trafficking.

## Discussion

In the past years, several image-based and high-throughput screens led to the identification of small molecules which affect membrane trafficking (Mishev et al., 2013). Among those, AMF-26/M-COPA, Golgicide A, Exo2, LG-186 and Tyrphostin AG1478 were identified as molecules displaying effects similar to BFA, but being more specific because they specifically target the ARF GEF GBF1 (Pan et al., 2008; Spooner et al., 2008; Saenz et al., 2009; Boal et al., 2010; Ohashi et al., 2012). In the present study, we initially intended to search for compounds that regulate the ER to Golgi transport of the Golgi-resident glycosylation enzyme ManII. Our study using a compound library composed of FDA-approved molecules as well as inhibitors of proteases, phosphatases and kinases revealed several compounds able to modulate ManII trafficking, either by inhibiting or accelerating ER to Golgi transport. Further studies will be necessary to clarify the biological activity of these molecules on ER to Golgi transport and more largely on secretory protein trafficking. We focused our attention on the previously studied Tyrphostin AG1478 as well as BML-265, which both prevent ManII transport to the Golgi, induce Golgi disassembly and prevent secretory protein transport. BML-265 and Tyrphostin AG1478 display a very close structure and are both annotated as Erlotinib analogs, being inhibitors of EGFR kinase activity. They share a quinazoline moiety with Erlotinib and other described inhibitors of receptor kinases (Fry et al., 1994; Ward et al., 1994; Stamos et al., 2002). Erlotinib as well as Gefitinib, Lapatinib and several Tyrphostins were present in our library but were not scored as molecules inhibiting the transport of ManII and/or affecting Golgi integrity. The similarity between BML-265 and Tyrphostin AG1478 in terms of structure and effects, and the absence of effects of other Erlotinib analogs, suggest that they both act on Golgi function independently of their ability to inhibit EGFR phosphorylation. Interestingly, other molecules known to target GBF1, such as Golgicide A, do not bear a quinazoline moiety (Saenz et al., 2009) and were not reported to inhibit EGFR phosphorylation.

Our results for BML-265 and Tyrphostin AG1478 as well as previous published work on Tyrphostin AG1478 (Pan et al., 2008) show that these small molecules induce Golgi disruption in human cells, but not in rodent cells. The differential effects of BML-265 and Tyrphostin AG1478 on human versus rodent cells are interesting in the point of view of their clinical use. EGFR is activated due to mutation and/or overexpression in diverse epithelial tumors and is associated with poor prognosis (Nicholson et al., 2001). Consequently, inactivation of EGFR signaling pathway is a target for cancer treatment. Erlotinib is given to patients sometimes in combination with cetuximab for the treatments of some cancers. Considering that mouse cells are sensitive only to the EGFR kinase inhibition activity of BML-265 and Tyrphostin AG1478, *in vivo* tests in mouse models would underestimate their toxic effects and fail to early capture clinically significant secondary effects that might arise in humans. The suggested target for these compounds is the *cis*-Golgi ARF GEF GBF1 since they exert BFA-like effects, but do not induce endosome tubulation. Nucleotide exchange activity of GBF1 is mediated by its catalytic Sec7 domain (Renault et al., 2003). The Sec7 domains of human and mouse GBF1 display 98% of similarity in their amino acid sequence. Even though the catalytic domain of GBF1 is highly conserved in human and mouse cells, this difference might be sufficient to modify the putative binding sites of BML-265 and Tyrphostin AG1478. In the present study, we showed that overexpression of human GBF1 prevents the effects of BML-265 on Golgi integrity. This result strongly suggests that GBF1 might be a target of BML-265. However, we cannot exclude indirect effects of the molecules on GBF1 neither the existence of other cellular upstream targets. The mode of interaction of these molecules with human GBF1 as well as the mechanisms of inactivation of GBF1 require further investigation.

## Data Availability Statement

All datasets generated for this study are included in the manuscript/Supplementary Files.

## Author Contributions

GB carried out the experiments and analyzed the data. NG carried out the experiments. ST and AL carried out high content screens. TJ and ED analyzed the high-content screening data. ED supervised the high content-screening. OK and GK helped in the analysis of the high content screening data. GB, FP, and ED wrote the manuscript. GB and FP designed the study.

## Conflict of Interest

OK and GK are cofounders of Samsara Therapeutics.

The remaining authors declare that the research was conducted in the absence of any commercial or financial relationships that could be construed as a potential conflict of interest.

## Supplementary Material

FIGURE S1 A dose-response analysis of 50 molecules led to the identification of 4 classes of molecules. HeLa cells stably expressing Str-KDEL_ManII-SBP-EGFP were incubated with serial dilutions of 50 selected molecules. Trafficking was induced by addition of biotin and phenotypic analysis was carried out using the approach developed for the screen. ‘ER-retained’ **(A)** and ‘Golgi-disrupted’ **(B)** scores were calculated and displayed on the graphs. Three independent replicates were performed and reported here (colored curves).

FIGURE S2 Effects of BML-265, AG1478 on different markers of intracellular compartments. **(A)** HeLa cells were incubated with the indicated molecules at 10 μM final for 1 h. After fixation, immunostaining using anti-TGN46 (left), anti-Sec24 (middle) or anti-GalT (right) antibodies was performed. Scale bar: 10 μm. **(B)** HeLa cells were incubated with the indicated molecules at 10 μM final for 1 h. Immunolabeling of early endosomes using an anti-EEA1 antibody, of the Golgi apparatus using an anti-Giantin antibody and of late/endosomes/lysosomes using an anti-LAMP1 antibody was performed. Scale bar: 10 μm.

FIGURE S3 BML-265 behaves as Erlotinib analog and inhibits EGFR phosphorylation but Erlotinib does not affect Golgi integrity. **(A)** HeLa cells were serum starved overnight and were treated with the indicated molecules at 10 μM for 90 min. Cells were then incubated with EGF at 50 ng/ml for 10 min. Phosphorylated EGFR (P-EGFR), total EGFR and actin (used as a loading control) were detected by immunoblot. **(B)** Scheme of the Erlotinib molecule. **(C)** HeLa cells were incubated with Erlotinib for 90 min at the indicated concentrations. The Golgi apparatus was stained using 3 different antibodies: anti-GalT (green), anti-GM130 (rouge), and anti-giantin (bleu). Scale bar: 10 μm.

MOVIE S1 The effects of BML-265 are reversible. HeLa cells stably expressing Str-KDEL_ManII-SBP-EGFP maintained in presence of biotin 40 μM to allow stable Golgi localization of ManII-SBP-EGFP. BML-265 at 10 μM was added at time 0. Time is indicated in min:sec. After 50 min, BML-265 was washed. Pictures were acquired every 30 s. Maximum projection of 11 z-slices is shown.
