# Supplementary figures and images for: BML-265 and Tyrphostin AG1478 Disperse the Golgi Apparatus and Abolish Protein Transport in Human Cells
Source: Front Cell Dev Biol. 2019 Oct 11;7:232. doi: 10.3389/fcell.2019.00232 (PMC6797785; doi:10.3389/fcell.2019.00232)

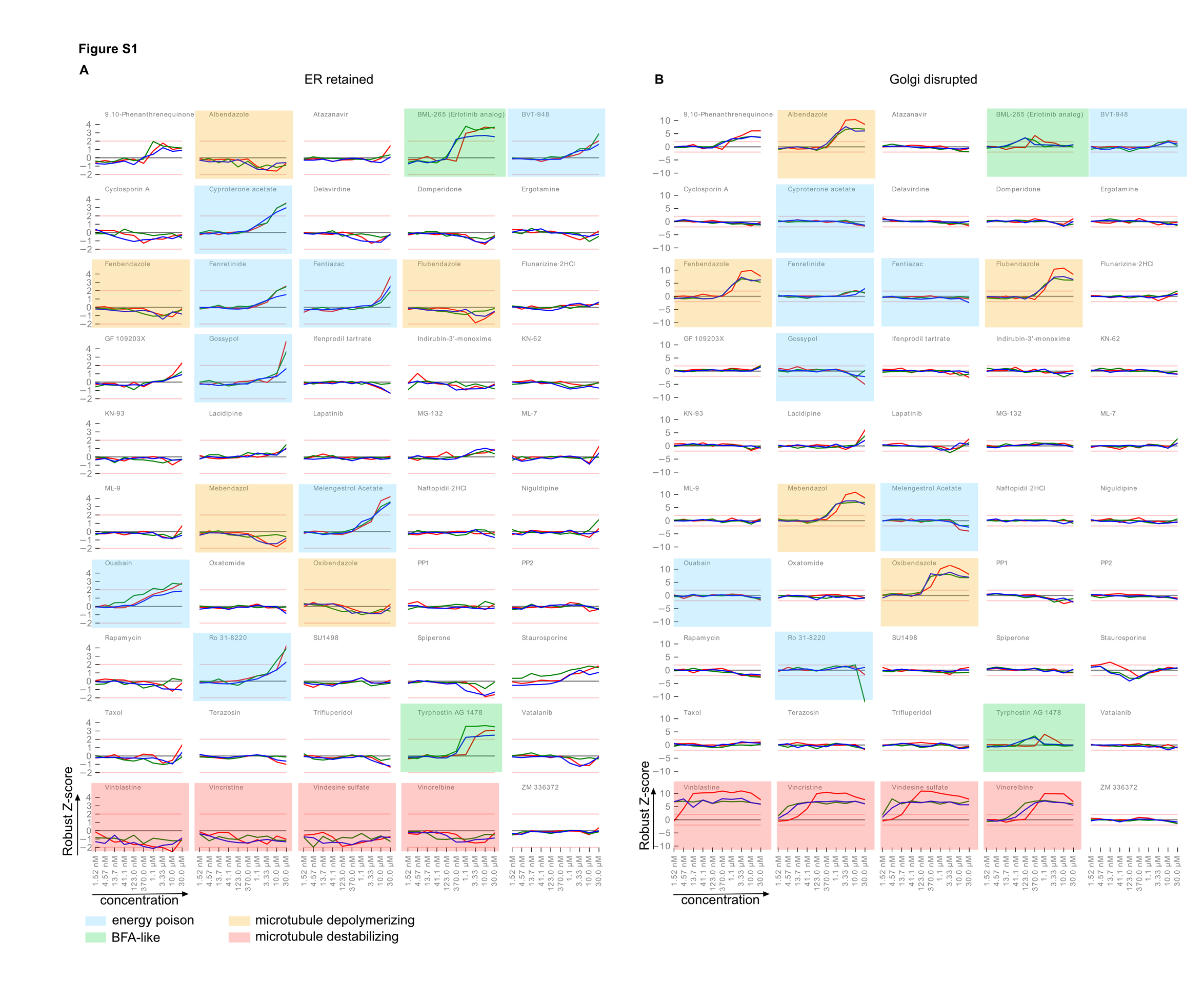

Supplement: FIGURE S1 — A dose-response analysis of 50 molecules led to the identification of 4 classes of molecules. HeLa cells stably expressing Str-KDEL_ManII-SBP-EGFP were incubated with serial dilutions of 50 selected molecules. Trafficking was induced by addition of biotin and phenotypic analysis was carried out using the approach developed for the screen. ‘ER-retained’ (A) and ‘Golgi-disrupted’ (B) scores were calculated and displayed on the graphs. Three independent replicates were performed and reported here (colored curves). [file Image_1.TIF]

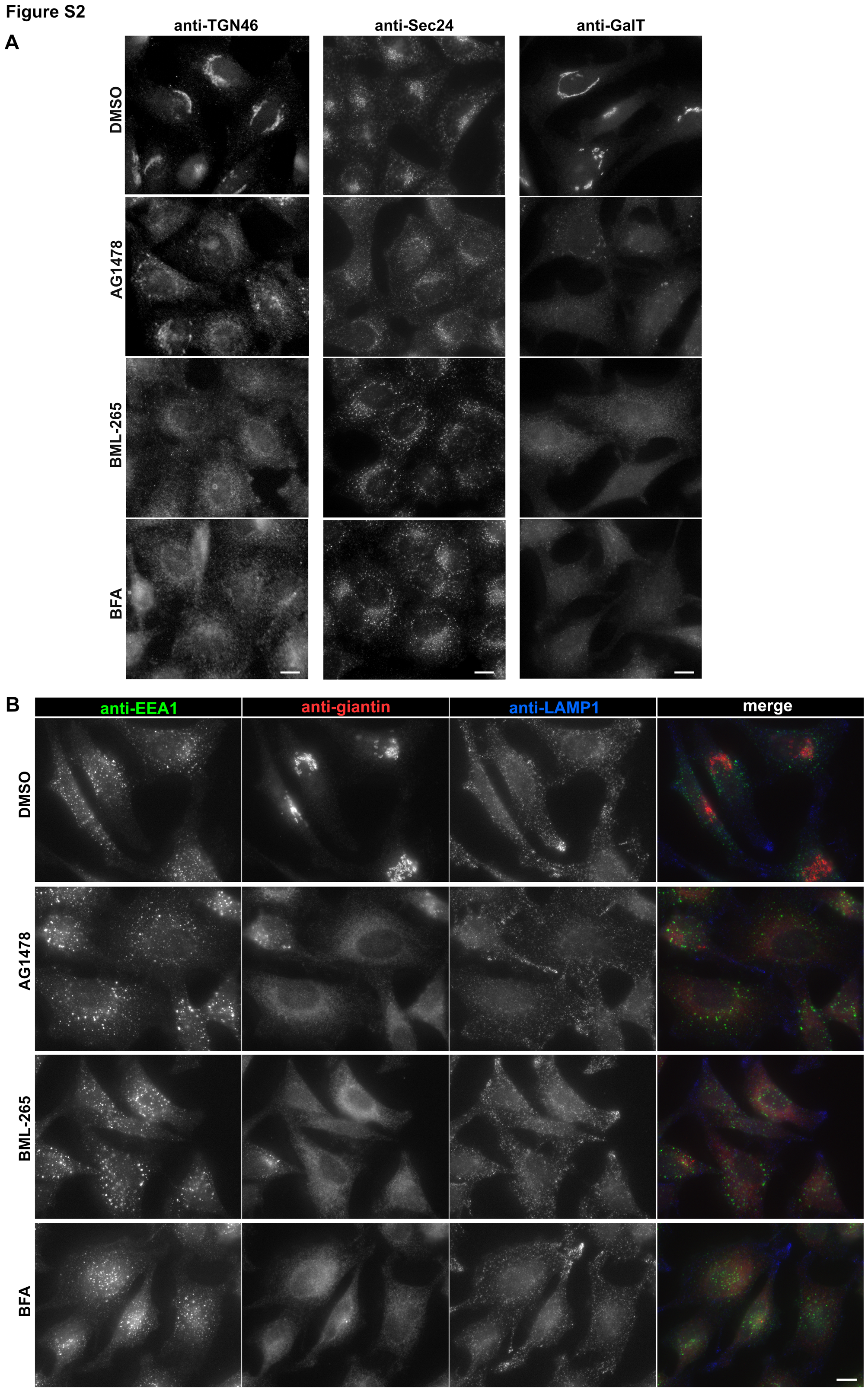

Supplement: FIGURE S2 — Effects of BML-265, AG1478 on different markers of intracellular compartments. (A) HeLa cells were incubated with the indicated molecules at 10 μM final for 1 h. After fixation, immunostaining using anti-TGN46 (left), anti-Sec24 (middle) or anti-GalT (right) antibodies was performed. Scale bar: 10 μm. (B) HeLa cells were incubated with the indicated molecules at 10 μM final for 1 h. Immunolabeling of early endosomes using an anti-EEA1 antibody, of the Golgi apparatus using an anti-Giantin antibody and of late/endosomes/lysosomes using an anti-LAMP1 antibody was performed. Scale bar: 10 μm. [file Image_2.TIF]

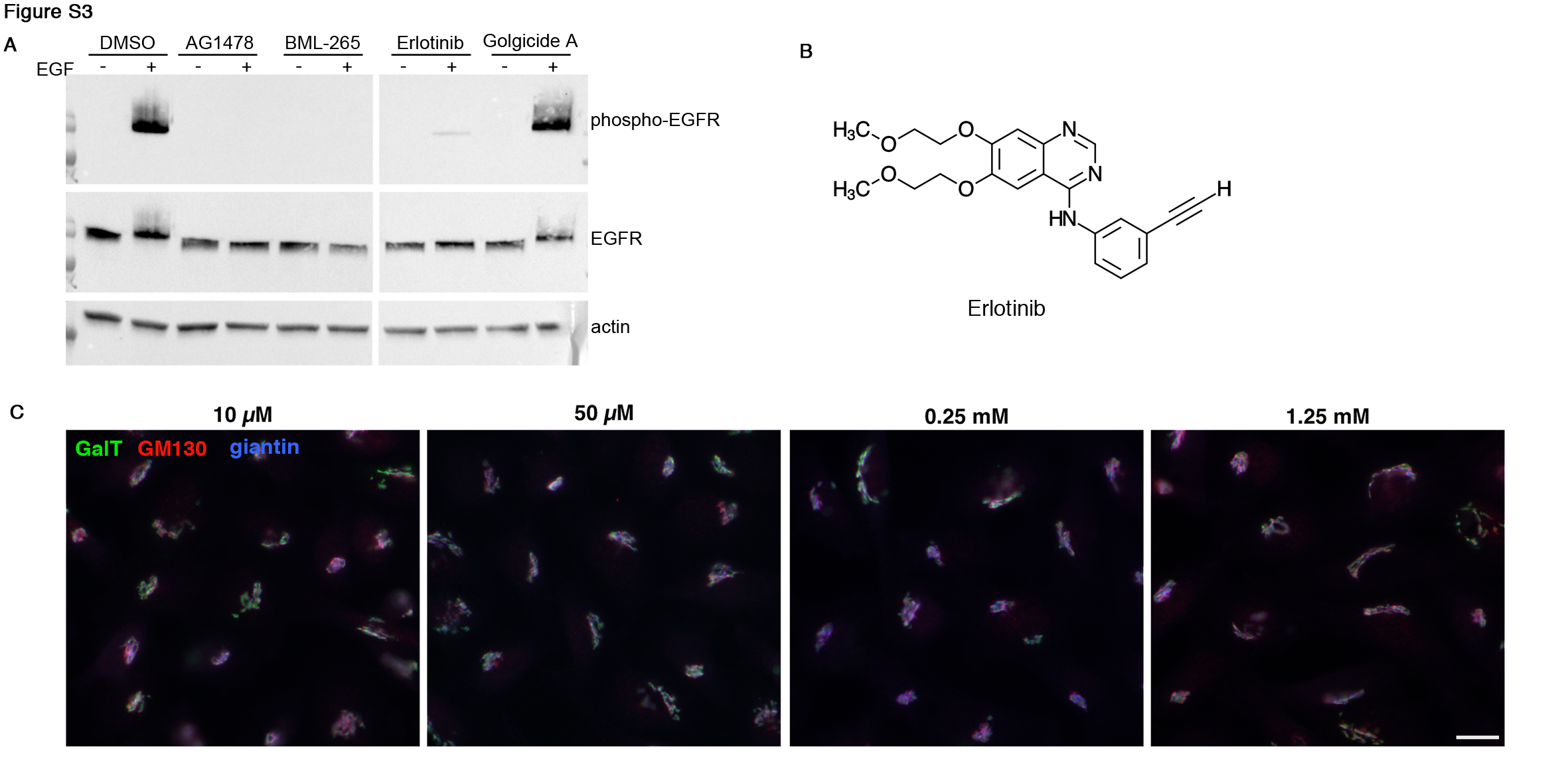

Supplement: FIGURE S3 — BML-265 behaves as Erlotinib analog and inhibits EGFR phosphorylation but Erlotinib does not affect Golgi integrity. (A) HeLa cells were serum starved overnight and were treated with the indicated molecules at 10 μM for 90 min. Cells were then incubated with EGF at 50 ng/ml for 10 min. Phosphorylated EGFR (P-EGFR), total EGFR and actin (used as a loading control) were detected by immunoblot. (B) Scheme of the Erlotinib molecule. (C) HeLa cells were incubated with Erlotinib for 90 min at the indicated concentrations. The Golgi apparatus was stained using 3 different antibodies: anti-GalT (green), anti-GM130 (rouge), and anti-giantin (bleu). Scale bar: 10 μm. [file Image_3.TIF]
